# Systemic inflammation in traumatic brain injury predicts poor cognitive function

**DOI:** 10.1002/iid3.577

**Published:** 2021-12-23

**Authors:** Wende Xu, Shenglei Yue, Peng Wang, Bin Wen, Xiaojue Zhang

**Affiliations:** ^1^ Department of Neurosurgery First People's Hospital of Tianshui Tianshui City Gansu Province PR China; ^2^ Department of Neurosurgery Sishui County Hospital Jining City Shandong Province PR China; ^3^ Department of Neurosurgery Lanling County People's Hospital Linyi City Shandong Province PR China

**Keywords:** cognitive function, systemic inflammation, traumatic brain injury

## Abstract

**Background:**

Traumatic brain injury (TBI) impairs cognitive function. Systemic inflammation plays important role in cognitive deficits. It remains unclear if systemic inflammation in TBI is associated with poor cognitive function.

**Methods:**

From January 2018 to December 2020, two groups of subjects were recruited: patients with TBI (*n* = 120), and healthy control (*n* = 120), followed up to 3 months. Blood was collected from TBI patients and healthy control, and serum inflammatory cytokines including interferon‐α (IFN‐α), interleukin‐1β (IL‐1β), IL‐6, IL‐8, IL‐10, and tumor necrosis factor‐α (TNF‐α) were measured at baseline and end of 3 months. Multivariate regression was used for analysis for the relationship between cognitive function and inflammatory cytokines.

**Results:**

Inflammatory cytokines were higher in patients with brain injury and remained high at end of 3 months. Some cytokines such as IFN‐α, IL‐1β, IL‐6, and TNF‐α were associated with worsening memory and predicted poor performance.

**Conclusion:**

Systemic inflammation in patients with TBI predicts poor cognitive function.

## 1 INTRODUCTION

Traumatic brain injury (TBI) caused by the external mechanical force is a diverse group of brain injuries such as skull or brain damage, strong rotations of head, or penetration of objects in the cranium.^1^ TBI varies in cause, severity, pathogenesis, and clinical outcome, which is the most common cause of long‐term disability and death among children and young adults.^2^ It is reported that about 5.48 million people suffer from severe TBI each year, leading to an enormous socioeconomic and healthcare burden.^3^ TBI may affect behavioral, cognitive, emotional, and psychological functions and life quality of patients.^4^, ^5^ Most importantly, TBI contributes to cognitive dysfunction in the diseases including chronic traumatic encephalopathy, Alzheimer's disease, and Parkinson's disease.^6^, ^7^, ^8^ New strategies including PC‐based rehabilitation or Virtual Reality Training (VRT) have been used to improve cognitive and behavioral skills in TBI patients.^9^, ^10^, ^11^

Inflammation can fight against invading pathogens and preserve healthy tissue while functions as a reactionary system to either aggravate or ameliorate tissue damage in pathological tissues.^12^, ^13^ It is suggested that inflammation is a central component of the secondary injury to the central nervous system (CNS) after TBI, and inhibition of inflammation is regarded as a promising target for TBI treatment.^14^ A previous study provided the evidence that following therapeutic inhibition of inflammasome activation obviously reduces innate immune activation and severe TBI.^15^ However, few studies were conducted on the inflammation resulting in secondary brain damage. In this study, we evaluated the systemic inflammation in TBI patients and predicted the relationship between inflammation and cognitive functions in TBI patients.

## 2 MATERIALS AND METHODS

### 2.1 Patients

A total of 120 patients with TBI (age: 36.56 ± 8.21 years; 54.76% males), who attended our hospital from January 2018 to December 2020, were enrolled in this study. In addition, 120 healthy persons (age: 37.73 ± 9.1 years; 52.38% males) were included in this study. These patients and healthy controls had blood samples prospectively collected at admission and after 30 days of follow‐up. This study was approved by the Ethics Committee of the First People's Hospital of Tianshui, and informed consent was obtained from all patients and healthy control participated in this study.

Inclusion criteria: neurological diagnosis of mild to moderate TBI; ability to sit for at least 20 min; presence of mild to moderate cognitive impairment.

Exclusion criteria: age older than 80 years; presence of disabling sensory alterations and frequent episodes of recurrent; other medical and psychiatric illness.

### 2.2 Enzyme‐linked immunosorbent assay (ELISA) assay

A total of 5 ml of venous blood was obtained from healthy controls and TBI patients, centrifuged at the speed of 3000 r/min for 10 min, and serum was collected. The expression levels of serum IFN‐α, IL‐1β, IL‐6, IL‐8, IL‐10, and TNF‐α were measured by ELISA kits (all from Merck‐Millpore) according to manufacturers.

### 2.3 Cognitive assessment

Montreal Cognitive Assessment (MoCA) was used to test cognitive function, which contained 30 items: short‐term memory (5 points); visuospatial abilities via clock drawing (3 points), and a cube copy task (1 point); executive functioning via an adaptation of Trail Making Test Part B (1 point), phonemic fluency (1 point), and verbal abstraction (2 points); attention, concentration, and working memory via target detection (1 point), serial subtraction (3 points), digits forward (1 point), and digits backward (1 point); language via confrontation naming with low‐familiarity animals (3 points), and repetition of complex sentences (2 points); and orientation to time and place (6 points).^16^

### 2.4 Data analysis

Data were analyzed using the Statistical Product and Service Solutions (SPSS) version 19.0 (SPSS, Inc.), and expressed as mean ± standard deviation (SD). A *p* < .05 was considered as statistically significant. Using SPSS, we performed the analysis of variance (ANOVA) to assess the difference at baseline and after 30 days of follow‐up. Multivariable logistic regression was used to calculate adjusted odds ratios with 95% confidence intervals (CI) to examine the relationship between the risk of cognitive impairment and inflammatory cytokines.

## 3 RESULTS

### 3.1 General characteristic of patients

General characteristic of TBI patients and healthy controls was listed in Table 1. No significant differences were found in participant number (*p* = .000), age (*p* = .1424), sex (*p* = .1082), and education (*p* = .8613) between TBI patients and healthy controls at baseline. Interval from TBI patients was 3 ± 0.58 months. Twenty‐eight cases of brain lesion occurred on cortical left, 6 cases on cortical right, 35 cases on subcortical left, and 51 cases on subcortical right.

**Table 1:** Characteristics for both healthy control group and TBI patient group

Items	Healthy control group	TBI patient group	*p* value
Participants (number)	120	120	.000
Age (years)	36.56 ± 8.21	37.73 ± 9.1	.1424
Education (years)	16.25 ± 1.66	16.16 ± 2.03	.8613
Gender			
Male	63	66	.1082
Female	57	54	.1157
Interval from TBI (mean in months)	0	3 ± 0.58	.0001
Brain lesion site/side			
Cortical left	0	28	.0001
Cortical right	0	6	.0001
Subcortical left	0	35	.0001
Subcortical right	0	51	.0001

Abbreviation: TBI, traumatic brain injury.

### 3.2 Changes of inflammation of TBI patients

The expression levels of proinflammatory cytokines, including IL‐1β, IL‐10, IL‐6, IL‐8, IFN‐α, and TNF‐α were detected in this study. As shown in Table 2, the levels of proinflammatory cytokines were significantly increased in TBI group compared to the control group (*p* < .001), indicating that TBI leads to inflammation in patients. Meanwhile, compared with TBI group, it was observed that the protein expressions of these proinflammatory cytokines induced by TBI were markedly reduced in the TBI group which was given a treatment after 30 days of follow up (*p* < .001), while the expressions were obviously higher that those in the healthy control group (*p* < .05). The results suggest that the inflammation is involved in TBI patients before or after treatment (Table 3).

**Table 2:** Inflammation between health control group and TBI patient group

Items	Healthy control group	TBI patient group	TBI patient group after 30 days of follow up	*p* value
IL‐6 (pg/ml)	42.29 ± 5.18	122 ± 7.85	122 ± 7.85	<.05
IL‐8 (pg/ml)	35.73 ± 2.62	302 ± 12.26	146 ± 10.38	<.05
IL‐10 (pg/ml)	34.33 ± 7.03	108.55 ± 10.37	75.45 ± 12.26	<.05
IL‐1β (pg/ml)	15.17 ± 1.26	28.87 ± 2.44	21.25 ± 2.49	<.05
TNF‐α (ng/ml)	11.06 ± 2.62	22.06 ± 4.37	16.45 ± 2.43	<.05
IFN‐α (ng/ml)	9.32 ± 1.35	18.24 ± 3.15	12.81 ± 3.66	<.05

Abbreviations: IFN‐α, interferon‐α; IL, interleukin; TBI, traumatic brain injury; TNF‐α, tumor necrosis factor‐α.

**Table 3:** Cognitive test and scale scores

Items	Healthy control group	TBI patient group	TBI patient group after treatment	*p* value
MoCA ≥15 (%)	99.36	95.62	98.35	<.05
MoCA ≥18 (%)	99.08	90.17	94.29	<.05
MoCA ≥21 (%)	89.37	63.86	80.33	<.05

Abbreviations: MoCA, Montreal Cognitive Assessment; TBI, traumatic brain injury.

### 3.3 Correlation between inflammation and cognitive function

First, we evaluated score distributions of the MoCA in TBI patients and healthy controls. The MoCA ≥15 points detected in healthy control (99.36%), TBI patients (95.62%), and TBI patients after treatment (98.35%). The MoCA ≥18 was 99.08%, 90.17%, 94.29% in healthy controls, TBI patients, and TBI patients after treatment, respectively. The MoCA ≥21 was 89.37%, 63.86%, 80.33% in above three groups.

Furthermore, the 95% CI for cognitive impairment and cytokines are presented in Table 4. Multivariate analysis showed that 95% CI of cytokines IL‐6, IL‐1β, TNF‐α, IFN‐α, and cognitive function was high, indicating a significant correlation between cognitive impairment and these cytokines. However, we observed a low 95% CI of cytokines IL‐8 and IL‐10 and cognitive function. It indicates systemic inflammation in patients with TBI predicts poor cognitive function.

**Table 4:** Relationship between inflammation and cognitive function

Items	95% CI
IL‐6	5.42
IL‐8	0.72
IL‐10	0.86
IL‐1β	36.94
TNF‐α	25.90
IFN‐α	4.29

Abbreviations: CI, confidence interval; IFN‐α, interferon‐α; IL, interleukin; TBI, traumatic brain injury; TNF‐α, tumor necrosis factor‐α.

## 4 DISCUSSION

Previous studies demonstrated that TBI initiates a secondary injury such as acute and chronic inflammatory events, thus aggravating outcome or promoting reparative processes.^16^ Another study suggested that inflammation is believed to participate in the pathophysiology of TBI.^17^ In a study in treatment strategies, it was demonstrated that a reduction in leukocyte infiltration into the post‐injured brain improves both histopathological and functional outcome measures.^18^ It is now well accepted that inhibition of inflammation can improve the prognosis of TBI, and it is effective in the recovery of patients with TBI.^19^ In this study, it is confirmed that systemic inflammation may predict patients with TBI.

The Mini‐Mental State Examination (MMSE) and MoCA are widespread and concise screening tools for the assessment of cognitive impairment that has a significant impact on the evaluation of age‐related cognitive decline.^20^ Recently, the MoCA has been developed to detect mild cognitive impairment with a score range 0–30 in China and other countries.^21^, ^22^, ^23^ In addition, MoCA can address orientation, drawing figures, processing speed, naming objects, memory, recall, attention, vigilance, repetition, verbal fluency, and abstraction.^24^ In the present study, it was suggested that the MoCA is effective to evaluate cognitive impairment.

Both clinical and experimental investigations have suggested that an elevated expression of cytokines including TNF‐α, transforming growth factor‐β (TGF‐β), and IL‐1β, IL‐6, and IL‐10 were found after TBI.^25^, ^26^, ^27^ Cytokines acted as classical neurotransmitters recruit additional blood‐borne neutrophils and monocytes into the injured tissue to activate the inflammatory cascade.^28^ Chronic inflammation induced by overexpression of IL‐6 or TNF‐α can promote the loss of GABAergic neurons in the hippocampus due to a reduction in synaptic inhibition.^29^, ^30^, ^31^ The expression of proinflammatory cytokine IL‐1β from peripheral blood mononuclear cells increases significantly 48–60 h after TBI and contributes to the inflammatory events that lead to neuronal loss.^32^, ^33^ Also, an upregulation in serum expression of TNF‐α, IL‐1β, IL‐6, IL‐8, and IL‐10 in TBI patients is found compared to the healthy control, and inflammation can be used to predict poor cognitive function.

## 5 CONCLUSIONS

This study suggests that inflammation may be a useful biomarker for patients with TBI, and its inhibition potentially leads to better cognitive and behavioral outcomes. Further studies are needed to assess how to inhibit inflammation to improve overall functional recovery and quality of life in TBI patients.

## CONFLICT OF INTERESTS

The authors declare that there are no conflict of interests.

## AUTHOR CONTRIBUTIONS

All authors have read and approved the manuscript. Guarantor of integrity of the entire study: Wende Xu. Study concepts: Wende Xu. Study design: Shenglei Yue. Definition of intellectual content: Shenglei Yue. Literature research: Peng Wang. Clinical studies: Peng Wang. Experimental studies: Bin Wen. Data acquisition: Xiaojue Zhang. Data analysis: Bin Wen. Statistical analysis: Xiaojue Zhang. Manuscript preparation: Wende Xu, Panyi Zhang. Manuscript editing: Wende Xu. Manuscript review: Wende Xu.
